# Dimethyl fumarate improves cognitive impairment by enhancing hippocampal brain-derived neurotrophic factor levels in hypothyroid rats

**DOI:** 10.1186/s12902-022-01086-4

**Published:** 2022-07-22

**Authors:** Haiyan Pan, Yanbo Wang, Xiaowei Wang, Ci Yan

**Affiliations:** 1 Department of Endocrinology, The Third Affiliated Hospital of Zhejiang Chinese Medicine University, Hangzhou, 310000 China; 2 Departments of Psychiatry, Affiliated Mental Health Center, Zhejiang University School of Medicine, Hangzhou, 310000 China

**Keywords:** Dimethyl fumarate, Hypothyroidism, Cognitive function, Brain-derived neurotrophic factor

## Abstract

**Background:**

Dimethyl fumarate (DMF) is an effective drug for multiple sclerosis and can improve the cognitive dysfunction caused by streptozotocin, but the effect on cognitive dysfunction caused by hypothyroidism is unclear.

**Methods:**

After the hypothyroidism rat model induced by propylthiouracil, we gave rats 25 mg/kg DMF by gavage. The body weight during model building and administration was recorded. The levels of T4 and T3 in serum were detected by an automatic biochemical analyzer. Morris water maze test was used to detect the effect of DMF on cognitive learning ability. The effect of DMF on Nissl bodies in the brain tissue was evaluated by Nissl staining. The mRNA and protein levels of BDNF in brain tissue were detected by quantitative reverse transcription-polymerase chain reaction and Western blot. The degrees of p-AKT/AKT and p-CREB/CREB in brain tissue were detected by Western blot.

**Results:**

After DMF treatment, the body weight of hypothyroid rats recovered, and the levels of T3 and T4 in the serum were ameliorated. DMF also reduced the escape latency and distance traveled, and increased the swim speed. The number of Nissl bodies and expression of BDNF, p-AKT/AKT, and p-CREB/CREB in the brain tissue were increased after DMF treatment.

**Conclusion:**

DMF improved the cognitive dysfunction of hypothyroid rats by increasing the level of BDNF in the brain tissue of hypothyroid rats.

**Supplementary Information:**

The online version contains supplementary material available at 10.1186/s12902-022-01086-4.

## Background

Hypothyroidism is a syndrome caused by insufficient thyroid hormone secretion [1]. Thyroid hormones have a vital impact on the development and normal work of the brain throughout life [2]. At the same time, thyroid hormones play an important role in cognition [3]. Clinical psychology studies have shown that the attention, mobility, memory and spatial ability of patients with hypothyroidism are significantly reduced, while the index of depression and anxiety is increased [4]. In addition, animal behavior studies have shown that the spatial memory ability of hypothyroid animals is impaired [5]. Although various degrees of cognitive impairment caused by hypothyroidism have been recognized by academia, the specific mechanism is still unclear.

Studies have suggested that cognitive dysfunction caused by hypothyroidism may be related to the down-regulation of Brain-derived Neurotrophic Factor (BDNF) [6, 7]. BDNF is a member of the neurotrophic factor, and it has a high content in the hippocampus and prefrontal cortex. BDNF can not only promote the growth and differentiation of neurons but also participate in the regulation of synaptic transmission and synaptic plasticity [8]. Animal experiments have shown that deleting BDNF in the broad forebrain regions of rats will cause damage to the hippocampus-dependent learning ability of rats [9]. And artificially increasing the level of BDNF can improve the spatial memory ability of rats [10, 11]. BDNF is also important for human brain function. Michael F Egan et al. found that BDNF val66met polymorphism is associated with human hippocampal-dependent memory impairment [12].

Fumaric acid esters (FAEs) are compounds that have antioxidant and anti-inflammatory effects in a variety of tissues and cells, and dimethyl fumarate (DMF) is the most biologically active compound in FAE [13]. DMF was used in the treatment of psoriasis [14]. In addition, DMF can also be used to treat multiple sclerosis [15]. DMF was first applied to the study of the nervous system. Isabel Lastres-Becker et al. reported the improvement of DMF on dyskinesia of neurodegenerative disease Parkinson’s mice [16]. Ludwig Kappos and his colleagues found that DMF inhibits the oxidative damage of nerve cells by activating the Nrf2 pathway, and maintains the integrity of nerve cell myelin [17]. It is worth noting that studies have shown that DMF reduces secondary degeneration after spinal cord injury by increasing the expression of BDNF [18]. In addition, DMF also has a certain alleviating effect on the spatial memory impairment of Alzheimer’s rats [19]. However, there is currently a lack of studies evaluating DMF on cognitive function after hypothyroidism. Considering the role of BDNF in hypothyroidism, we speculate that DMF may play a role in hypothyroidism by affecting the expression of BDNF.

Therefore, we constructed a rat model of hypothyroidism induced by propylthiouracil (PTU) to observe the effects of DMF on the behavior of hypothyroidism rats in the Morris water maze, serum thyroid hormone levels and BDNF, p-AKT/AKT, p-CREB/CREB expression in the hippocampus, in order to clarify the effect of DMF on the learning and memory ability of hypothyroid rats and explore its possible mechanism.

## Methods

### Animals

Thirty male Sprague–Dawley rats (8 weeks old, 215 ± 20 g) were purchased from Shanghai Jihui Laboratory Animal Care Co., Ltd. (SCXK (Hu) 2017–0012). All rats were kept in an environment where the ambient temperature is maintained at 22–23 °C, the relative humidity is 45–50%, and the light cycle is 12/12 hours. All rats were fed adaptively for 1 week, and they were free to eat and drink. Those with normal drinking and eating were included in the experiment.

The construction of the PTU rat model referred to the previous literature [20]. Rats were randomly divided into the control group (*n* = 10) and the model group (*n* = 20). The rats in the control group drank water normally, while the rats in the PTU group drank tap water containing 0.05% PTU [21] (IP0420, Solarbio, China). After 28 days, the rats in the model group were randomly divided into the PTU group (*n* = 10) and the PTU + DMF25 group (*n* = 10). The rats in the control group and the PTU group were given saline once a day, while the rats in the PTU + DMF25 group were given 25 mg/kg DMF (ID0320, Solarbio, China) once a day for 14 days [22]. The body weight of each group of rats was measured weekly.

### Morris water maze test

The learning and memory abilities of rats are tested through the Morris water maze test [23]. The diameter of the maze was 1.6 m and the height was 50 cm. The pool water was dyed black with food coloring. The water depth was 30 cm, and the water temperature was 22–23 °C. The experiment was divided into two parts: positioning navigation and space search. In the positioning navigation experiment, the rats received 4 days of training, 4 times a day. The specific training was as follows: each time before entering the water, put the rat on the underwater platform to adapt for 30 seconds, record the swimming distance of the rat from the four quadrants and different entry points to the platform within 60 seconds, and the average of the 4 results was the final grade. On the fifth day, the space search experiment was carried out, the platform was removed, and the rats were placed into the water from the entry point in the opposite quadrant of the platform, facing the wall of the pool, and the swimming trajectory of the rats within 60 seconds was recorded and analyzed.

### Specimen collection and preparation

After the water maze experiment, the rats were fasted for 12 hours but were allowed to drink water freely. After the rats were anesthetized, blood was taken from the abdominal aorta, and the collected blood was centrifuged to obtain serum. The content of T3 and T4 in serum was measured by an automatic biochemical analyzer (C1600, Abbott, USA) within 48 hours. The rats were euthanized with an overdose of sodium pentobarbital, the brain tissues of the rats were separated, a part of the brain tissue was fixed in 4% paraformaldehyde, paraffin-embedded and sectioned, and the rest was stored in a refrigerator at − 80 °C for Western blot and quantitative reverse transcription-polymerase chain reaction (qRT-PCR).

### Nissl staining

After the paraffin sections (4 μm) were conventionally deparaffinized and hydrated, they were reacted with Nissl staining solution (G1036, Wuhan Google Biotechnology Co., Ltd., China) in an oven at 60 °C for 20 min. After washing the sections with distilled water, the sections were dried in an oven at 60 °C. Finally, the sections were routinely dehydrated, transparent, and sealed [24]. The number of Nissl bodies at the same site in each group of rats under a field of view was counted. And the average thickness of the granular layer was measured at five random positions in CA1 of each slice in each group under the same field of view [25] (Per × 400 field).

### qRT-PCR

Total RNA from rat brain tissue was extracted by a total RNA extraction kit (R1200, Solarbio, China). Total RNA was reverse transcribed into cDNA with the help of a reverse transcription kit (CW2569, cwbiotech, China). Then the SYBR Green qPCR kit (CW2601, cwbiotech, China) was used for qPCR. β-actin was employed as an internal control. The primers were listed as follows: BDNF, forward: 5′- GGCAGGCTTTGATGAGACCG-3′ and reverse: 5′-TCACCTGGTGGAACTCAGGGT-3′; β-actin, forward: 5′-AACCTTCTTGCAGCTCCTCC-3′ and reverse: 5′-TACCCACCATCACACCCTGG-3′. Relative expressions of BDNF were analyzed by a Real-Time PCR Detection system (CFX96, Bio-rad, USA) with the 2^-ΔΔCt^ method [26].

### Western blot

Western blot was performed as previously described [27]. The protein sample was collected from rat brain tissue by RIPA lysate (P0013D, Beyotime, China), PMSF (ST506, Beyotime, China) and protease inhibitors (60,237, Beyotime, China). Then the protein was sequentially quantified by the BCA kit (pc0020, Solarbio, China). The protein was separated with 10% separating gel, and then the protein was transferred to the PVDF membrane (10,600,023, GE Healthcare Life, USA). Before incubating the primary antibody, the membrane needed to be blocked with 5% skimmed milk. Primary antibodies include anti-BDNF (ab108319, Abcam, UK), p-AKT (20 t9742, Affinity, China), AKT (59z1942, Affinity, China), CREB (36v1551, Affinity, China), p-CREB (58y21722, Affinity, China) antibody and anti-β-actin antibody (ab8226, Abcam, UK). After incubating with the primary antibody overnight at 4 °C, the membrane reacted with the secondary antibody goat anti-rabbit (ab205718, Abcam, UK) or goat anti-mouse (ab6789, Abcam, UK) at room temperature for 2 hours. The membrane was developed on a chemiluminescence instrument (610020-9Q, Qinxiang, China) with an ECL luminescence reagent (C510045, Sangon, China). β-actin was used as the internal control.

### Statistical analysis

Data were analyzed by SPSS 16.0 (SPSS, Chicago, USA) and represented as mean ± standard deviation. One-way analysis of variance was used for measurement data among multiple groups, and Tukey test was used for comparison between groups. Kruskal-Wallis H test was used for results of uneven variance. *P* < 0.05 was accepted to be statistically significant.

## Results

### Effect of DMF treatment on body weight and thyroid level in rats with hypothyroidism

**Fig. 1:**
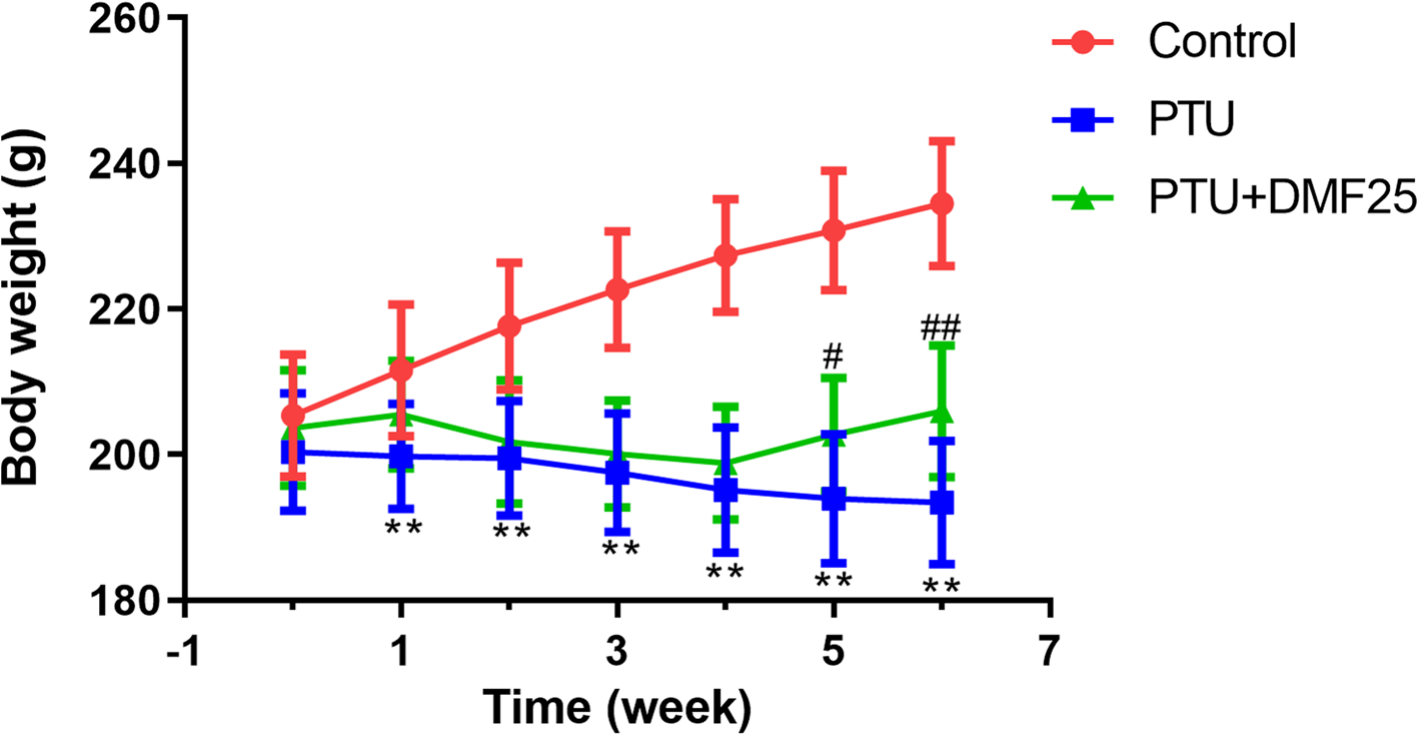
Comparison of the body weight increment among three groups ($\overline{x}$ ±s, *n* = 10). ^**^P < 0.01 compared with the control group, ^##^*P* < 0.01 compared with the PTU group

**Fig. 2:**
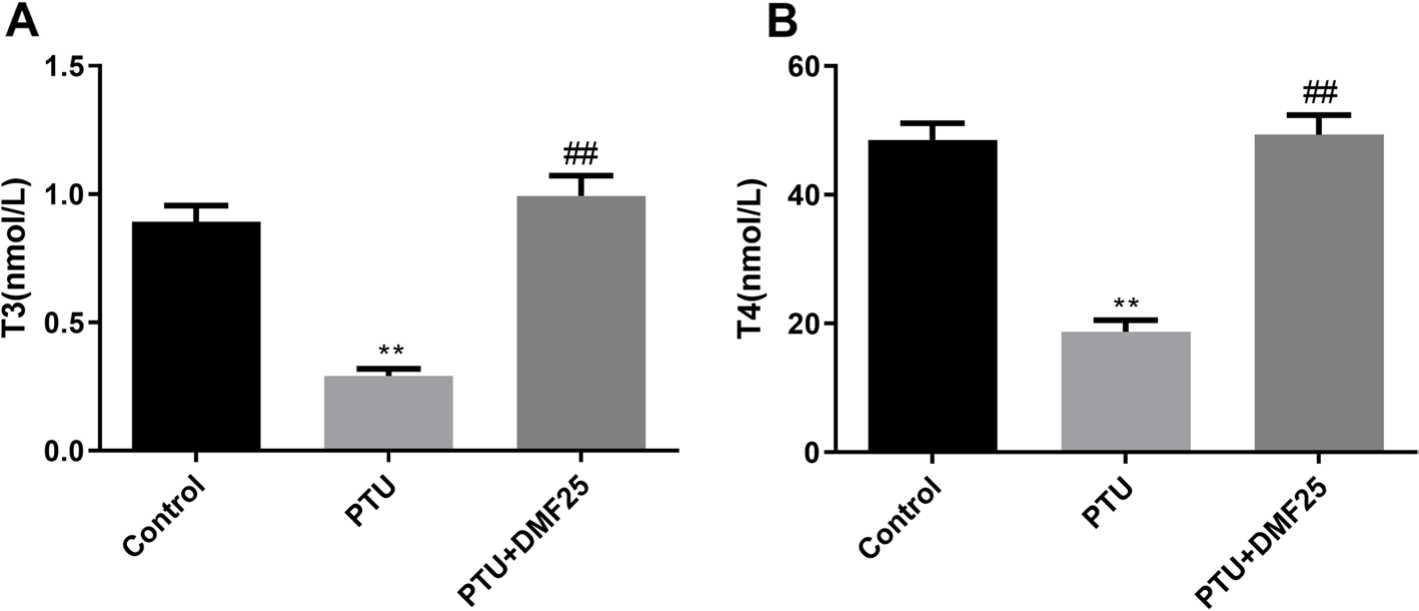
Comparison of serum of thyroid-related hormones levels among three groups ($\overline{x}$ ±s, *n* = 10). **A** The content of T3; **B** the content of T4. ^**^P < 0.01 compared with the control group, ^##^*P* < 0.01 compared with the PTU group

After the SD rats were experimentally fed for 1 week, the rats were divided into groups. At this time, there was no significant difference in the body weight of rats in each group (Fig. 1). During the 4 weeks when the rats were treated with 0.05% PTU, the weight of the rats was significantly lower than that of the control rats (Fig. 1, *P* < 0.01). However, after DMF treatment, the trend of weight loss in PTU group rats was reversed (Fig. 1, *P* < 0.05). In addition, the levels of T3 and T4 in the serum of rats with PTU-induced hypothyroidism were also significantly lower than those in the control group (Fig. 2, *P* < 0.01). However, the treatment of DMF restored the levels of T3 and T4 in the serum of rats in the PTU group to normal (Fig. 2, *P* < 0.01).

### Effect of DMF on the learning ability of hypothyroid rats

**Fig. 3:**
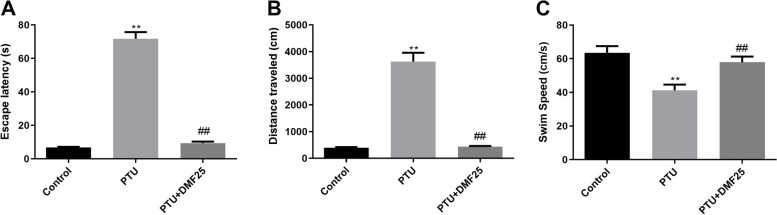
Comparison of the escape latency ($\overline{x}$ ±s, *n* = 10). **A** and distance traveled; **B** to reach the platform and the swim speed (**C**) between three groups in the Morris water maze test. ^**^P < 0.01 compared with the control group; ^##^*P* < 0.01 compared with the PTU group

We evaluated the effect of DMF on the learning and memory abilities of hypothyroid rats through the Morris water maze. As shown in Fig. 3, the escape latency of rats in the PTU group was much higher than that of the control group, while DMF treatment can effectively reduce the escape latency of hypothyroid rats to a normal level (*P* < 0.01). Not only that, compared with the control group, rats in the PTU group swam longer and had a slower swimming speed, but these problems can be improved by DMF treatment (*P* < 0.01).

### DMF increased the number of Nissl bodies in the hippocampal CA1 area of hypothyroidism rats

**Fig. 4:**
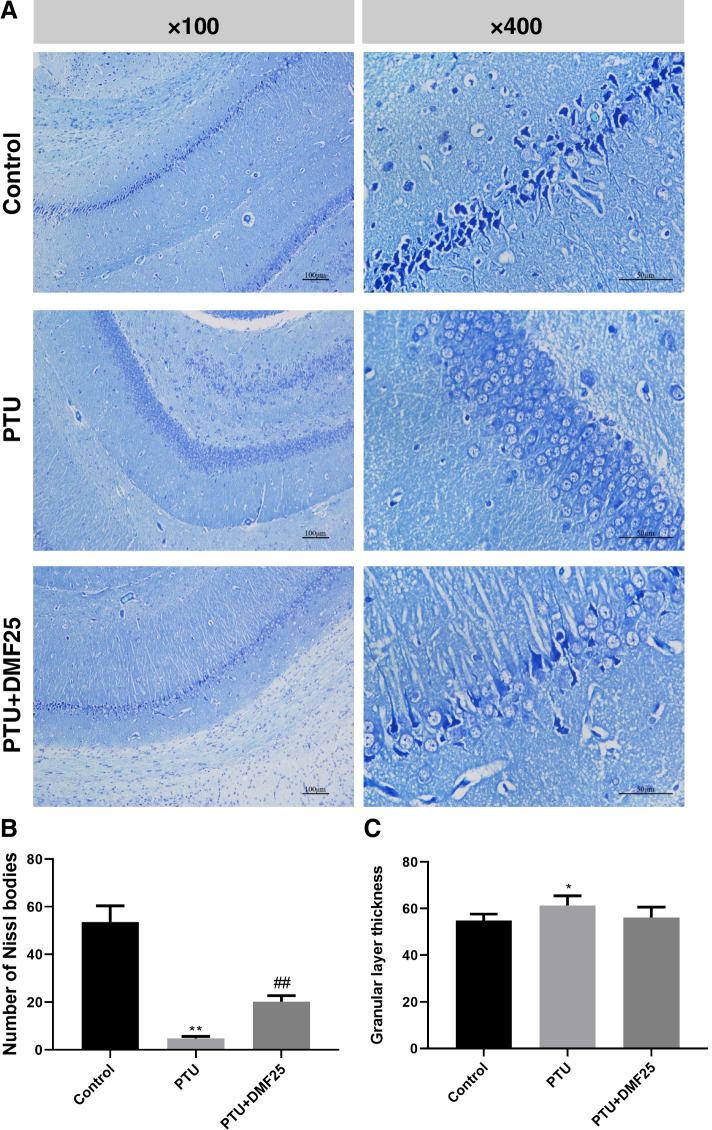
Effect of DMF on hippocampal neurons of rats (*n* = 5). **A** Representative images showing Nissl bodies in the hippocampal CA1 (× 100, Scale bar = 100 mm; × 400, Scale bar = 50 mm.); **B** Quantitation of pyramidal cells in the CA1 hippocampal region ($\overline{x}$ ±s). The number of Nissl bodies at the same site in each group of rats under a field of view was counted; **C** The thickness of granular layer of CA1 region of the hippocampus; The average thickness of granular layer was measured at five random positions in CA1 of each slice in each group under the same field of view. ^**^P < 0.01 compared with the control group; ^##^*P* < 0.01 compared with the PTU group

Nissl body is a small triangular or elliptical mass distributed in the cytoplasm of nerve cells, which can be stained blue-purple by Nissl staining solution. The disappearance of the Nissl body is an important indicator of nerve cell damage. From Fig. 4, we can see that the Nissl body in the control group is very complete and the neurons were closely aligned, while the number of Nissl bodies in the PTU group is significantly less than that in the control group (*P* < 0.01). Compared with the PTU group, the number of Nissl bodies in the PTU + DMF25 group was increased (*P* < 0.01), and the neurons were arranged relatively neat and close. Besides, the granular layer was significantly thickened in the PTU group than it was in the control group (*P* < 0.05). After DMF treatment, the thickness of the granular layer was reduced but not significantly different compared to the PTU group (*P* > 0.05).

### DMF increased the mRNA and protein level of BDNF in the hippocampal CA1 area of hypothyroid rats

**Fig. 5:**
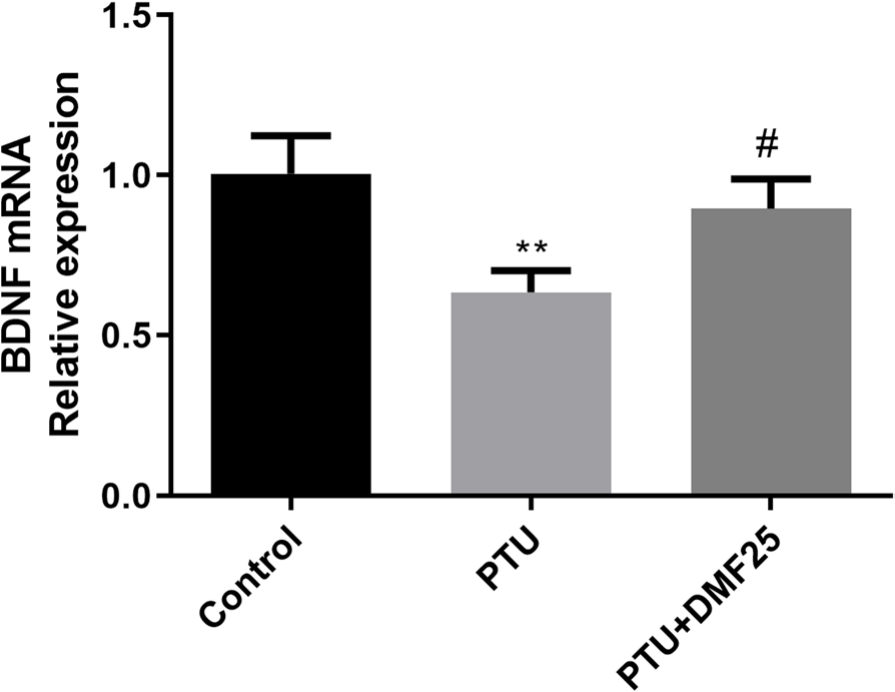
Effect of DMF on BDNF mRNA expression in hippocampal CA1 region of hypothyroidism rats ($\overline{x}$ ±s, *n* = 3). ^**^P < 0.01 compared with the control group. ^#^*P* < 0.01 compared with the PTU group

**Fig. 6:**
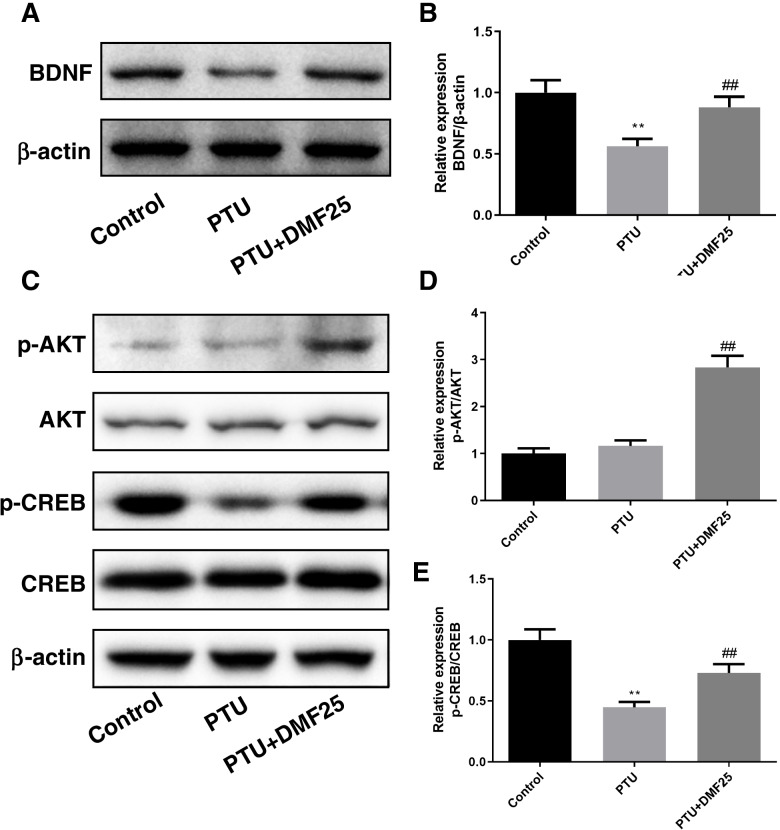
Effect of DMF on BDNF, p-AKT/AKT, p-CREB/CREB protein expression levels in hippocampal CA1 region of hypothyroidism rats ($\overline{x}$ ±s, *n* = 3). **A** The protein expression of BDNF; **B** the relative expression of BDNF protein; **C** the protein expression of p-AKT/AKT and p-CREB/CREB; **D** the relative expression of p-AKT/AKT; **E** the relative expression of p-CREB/CREB. ^**^P < 0.01 compared with the control group. ^##^*P* < 0.01 compared with the PTU group. Corresponding uncropped full-length gels and blot can be saw in the supplementary information

By detecting the expression of BDNF mRNA and protein in the brain tissue of each group of rats (Figs. 5, 6A, and B *P*< 0.01), we found that PTU-induced hypothyroidism rats express less BDNF in the brain tissue (*P* < 0.01). But the decrease of BDNF expression in rat brain tissue caused by PTU can be reversed by DMF treatment (*P* < 0.05, *P* < 0.01).

DMF increased the levels of p-AKT/AKT and p-CREB/CREB in the hippocampal CA1 area of hypothyroid rats.

Western blot was used to measure the levels of p-AKT/AKT and p-CREB/CREB (Fig. 6C). In the PTU group. The level of p-AKT/AKT was not significantly different compared to the control group (*P* > 0.05), and DEM treatment was significantly upregulated compared to the PTU group (*P* < 0.01) (Fig. 6D). The degree of p-CREB/CREB was markedly decreased in the PTU group compared to the control group (*P* < 0.01), while it was notably improved in the PTU + DEM25 group (*P* < 0.01) (Fig. 6E).

## Discussion

Thyroid hormones are essential for the normal development of the mammalian brain [28]. Mounting studies have shown that thyroid hormone deficiency during brain development can lead to abnormal brain structure and dysfunction, which seriously affects learning and memory functions [29, 30]. At present, the treatment of hypothyroidism mostly uses thyroid hormone replacement therapy [31]. Thyroid hormones mainly include T3, T4, and TSH. Clinically, hypothyroidism is usually diagnosed by detecting the levels of T3 and T4. The body weight, serum T3 and T4 levels decreased significantly in PTU-induced rats, and after DMF treatment, they were reversed. It suggested that DMF can improve hypothyroidism. Graciela Freitas Zarbato et al. found that the anti-inflammatory and antioxidant effects of DMF were exerted in experimental sepsis rats. In addition, they also found that DMF can improve cognitive impairment after bacterial sepsis [32]. Coincidentally, Sofia P das Neves and others also found that DMF can enhance the cognitive ability of mice with experimental autoimmune encephalomyelitis [33]. Therefore, our study used the classic Morris water maze experiment to evaluate the cognitive function of rats in each group, and found that DMF has the same alleviating effect on the cognitive impairment of PTU-induced hypothyroid rats. Besides, we found through Nissl staining that DMF can significantly improve the pathological morphology of neurons in the CA1 region of the hippocampus of hypothyroid rats. Similar to our conclusion, Xiaowen Hou et al. also found that DMF has a repairing effect on neuronal damage in the hippocampus CA1 area of rats with middle cerebral artery occlusion [34].

BDNF plays an important role in the survival, differentiation, migration of neurons, the development of axons and dendrites of new neurons, and the formation of synapses, in particular, it shows advantages for protecting memory functions [35]. When simulating learning-related signals, the expression of BDNF in the pre- and post-synaptic membranes will increase, which means that BDNF is one of the key proteins in learning and memory [36]. And BDNF plays a neuroprotective and growth-promoting role in damaged neuronal [37]. Wu’s study suggested that BDNF could inhibit autophagy to play a neuroprotection function [38]. Scientists working on neuronal damage in intracerebral hemorrhage found the TrkB/Akt signaling was important in neuronal protection [39]. Moreover, Liu’s team studied n-3 docosapentaenoic acid’s neuroprotection and found that it could activate BDNF/TrkB-PI3K/AKT signaling to protect neurons from neuroinflammation [40].

At present, the research on the regulation of cognitive function by DMF has mostly focused on the Nrf2 pathway and other oxidative stress pathways [22, 32, 41]. This is because neurons are extremely sensitive to the damage of reactive oxygen species. When reactive oxygen species are generated excessively, the oxidative stress response is enhanced, which can damage the nucleic acids, proteins, and lipids on the inner membrane of neuronal mitochondria, causing cell damage or death, and causing cognition disfunction [42, 43]. However, our study found that DMF treatment was able to promote the expression level of BDNF in the hippocampus of hypothyroid rats, which may be one of the biological mechanisms by which DMF improves memory function. In the study of Mohammad Saied Salehi and others, DMF can promote the expression of BDNF in epidermal neural crest stem cells [44]. The regulatory effect of DMF on BDNF has also been confirmed to improve the depressive behavior of rats and the secondary degeneration after spinal cord injury [18, 45]. In addition, a study found that DMF can enhance p-Akt and its downstream targets CREB and BDNF that they are neuroprotective [46]. In our study, DMF treatment can increase the levels of p-AKT, p-CREB and BDNF in the brain tissue of hypothyroid rats, which suggested that the regulation of DMF on p-AKT/p-CREB/BDNF can also improve the cognitive dysfunction of hypothyroid rats. Pallavi Mishra’s team found that oral T4 significantly increased p-Akt/Akt levels in rat hearts [47]. It suggested that the improvement of cognitive dysfunction by DMF might be associated with the up-regulation of T4, which warrants further exploration in future studies. Certainly, this study investigated the effects of DMF on improving memory ability and neuronal damage in hypothyroid rats, and made a preliminary exploration of its biological mechanism, about the biological action mechanism of DMF on promoting BDNF secretion will be deeply studied in the future.

## Conclusion

In general, we constructed a hypothyroid rat model, which proved that DMF can advance the levels of T3 and T4 in serum, improve cognitive behavior of hypothyroid rats to a certain extent, ameliorate hippocampal neuronal injury, raise the levels of BDNF, p-AKT/AKT, and p-CREB/CREB protein. However, we have only initially studied the effect of DMF on the expression of BDNF, and the regulation of its specific signaling pathway has not yet been clarified.

## Supplementary Information

**Additional file 1.**

## Data Availability

All data generated or analyzed during this study are included in this article.

## Abbreviations

BDNF: Brain-derived Neurotrophic Factor
FAEs: Fumaric acid esters
DMF: dimethyl fumarate
PTU: propylthiouracil
