# Supplementary material for: Dimethyl fumarate improves cognitive impairment by enhancing hippocampal brain-derived neurotrophic factor levels in hypothyroid rats
Source: BMC Endocr Disord. 2022 Jul 22;22:188. doi: 10.1186/s12902-022-01086-4 (PMC9306081; doi:10.1186/s12902-022-01086-4)

The details of all the supplementary files

The original blot of Figure 6A

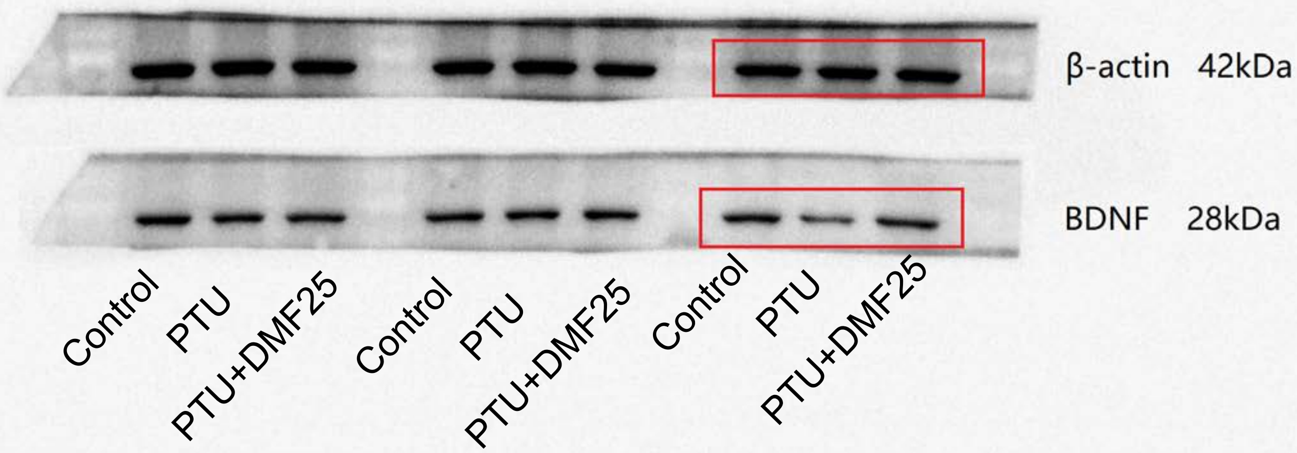

The original blot of Figure 6C

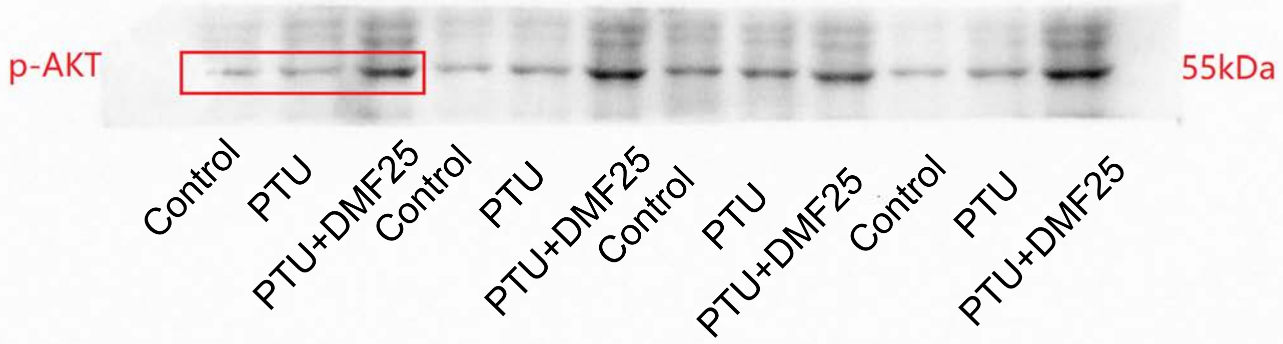

The original blot of Figure 6C

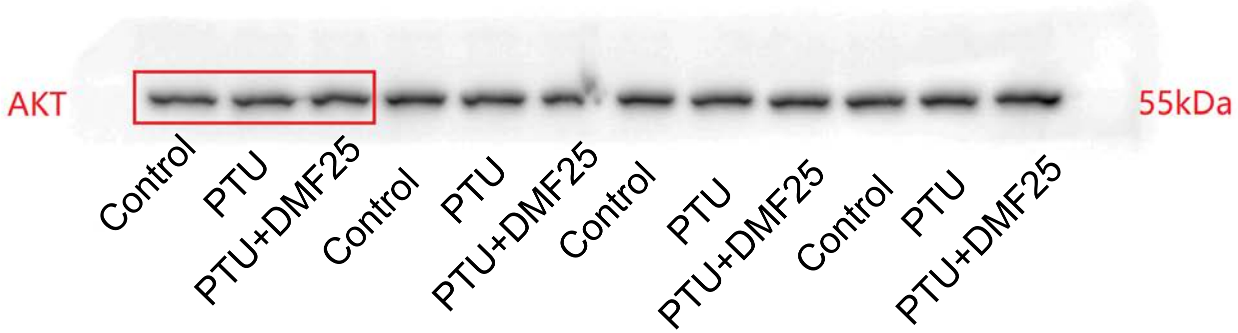

The original blot of Figure 6C

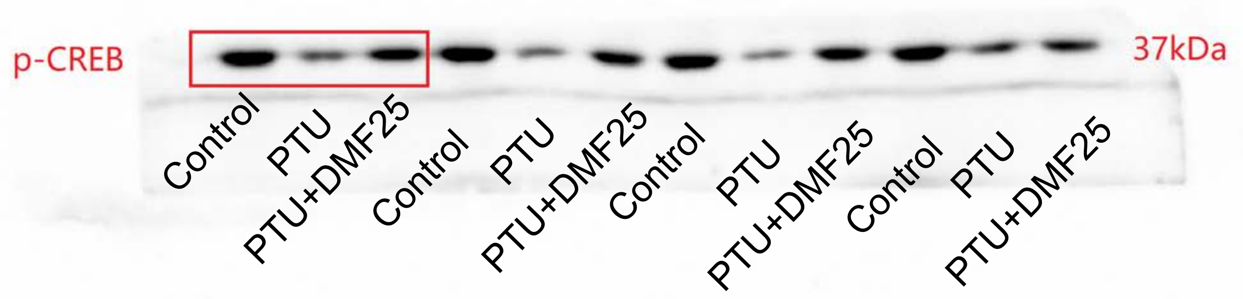

The original blot of Figure 6C

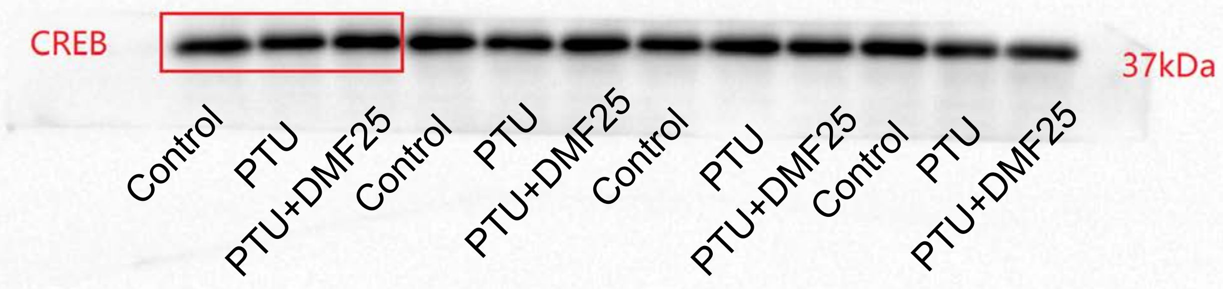

The original blot of Figure 6C

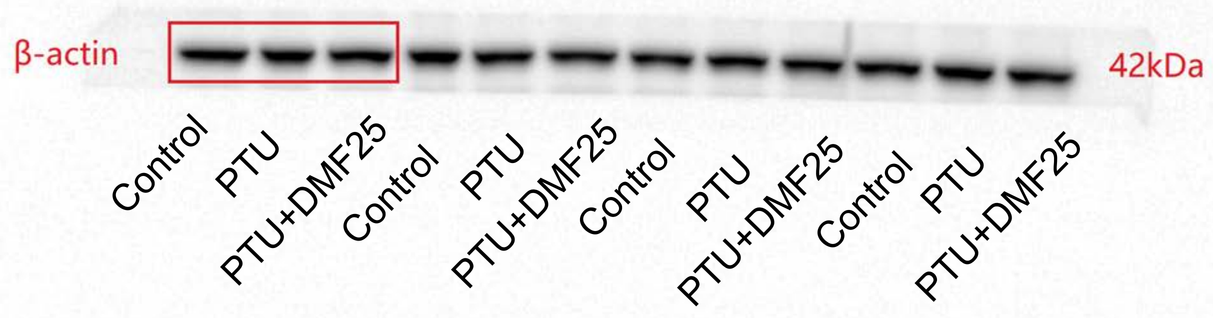

Supplement: Supplementary file 1 — Additional file 1. [file 12902_2022_1086_MOESM1_ESM.zip › The details of all the supplementary files.pdf]
