# Supplementary figures and images for: Dimethyl fumarate improves cognitive impairment by enhancing hippocampal brain-derived neurotrophic factor levels in hypothyroid rats
Source: BMC Endocr Disord. 2022 Jul 22;22:188. doi: 10.1186/s12902-022-01086-4 (PMC9306081; doi:10.1186/s12902-022-01086-4)

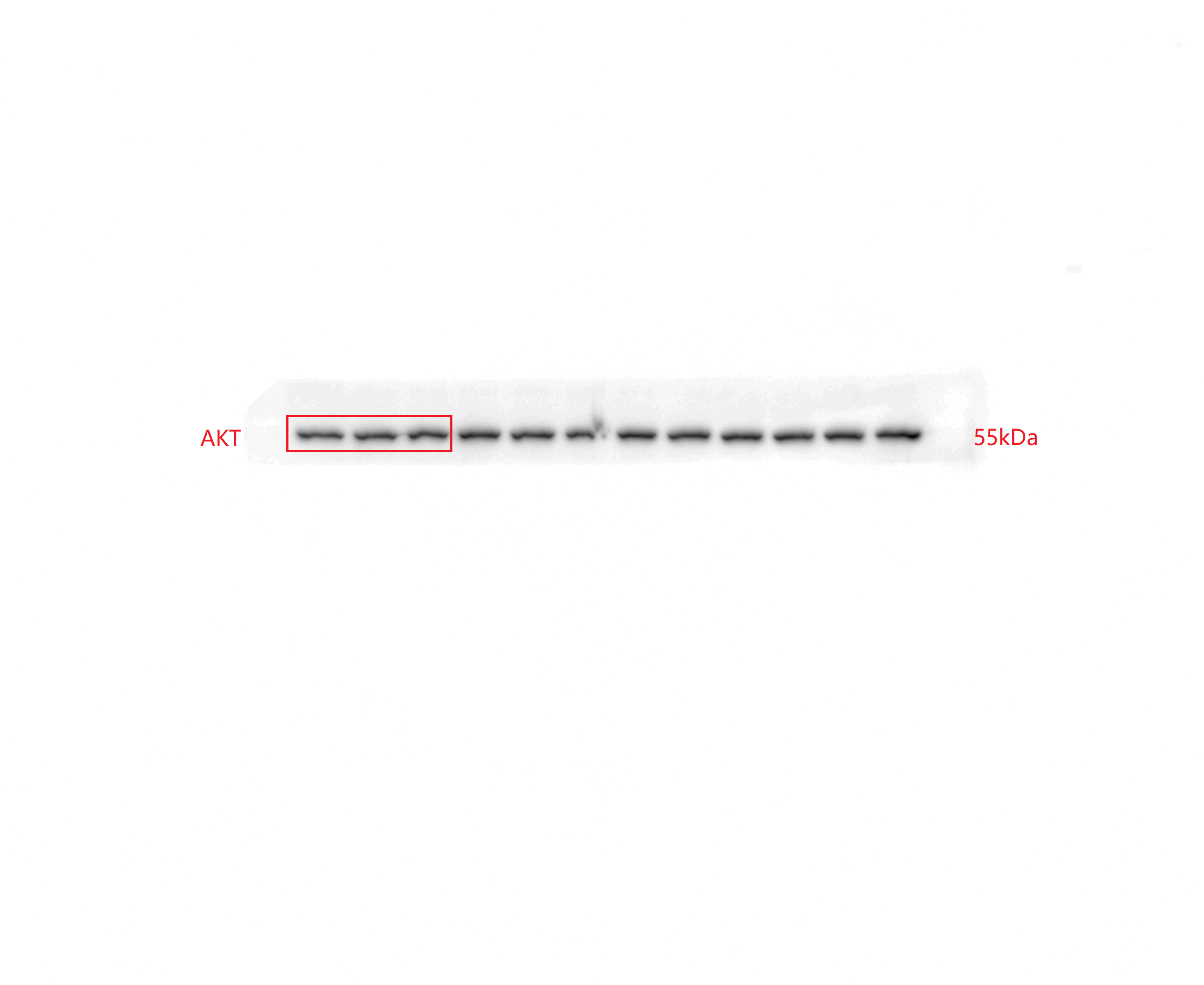

Supplement: Supplementary file 1 — Additional file 1. [file 12902_2022_1086_MOESM1_ESM.zip › AKT.tif]

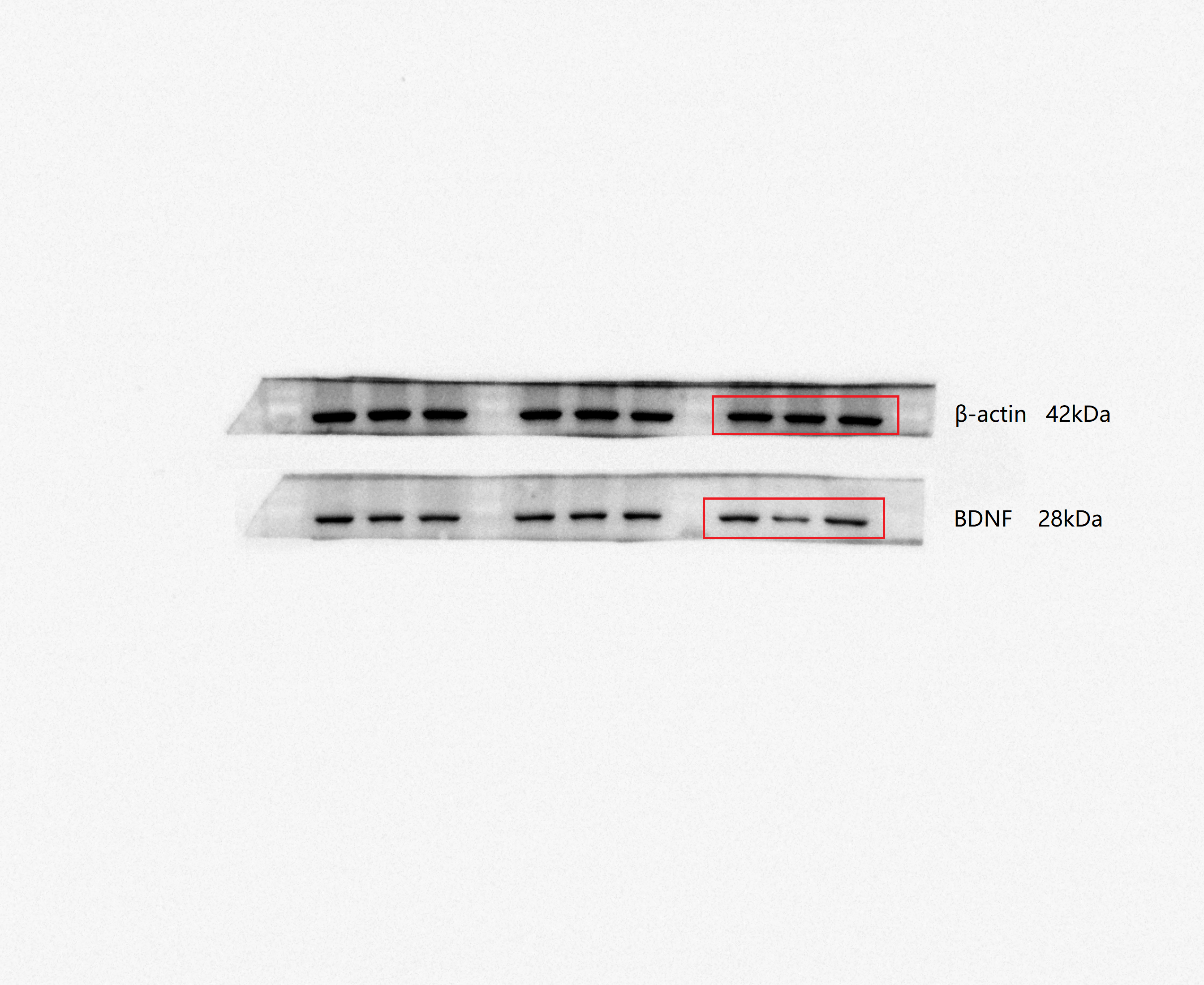

Supplement: Supplementary file 1 — Additional file 1. [file 12902_2022_1086_MOESM1_ESM.zip › BDNF and beta-actin.tif]

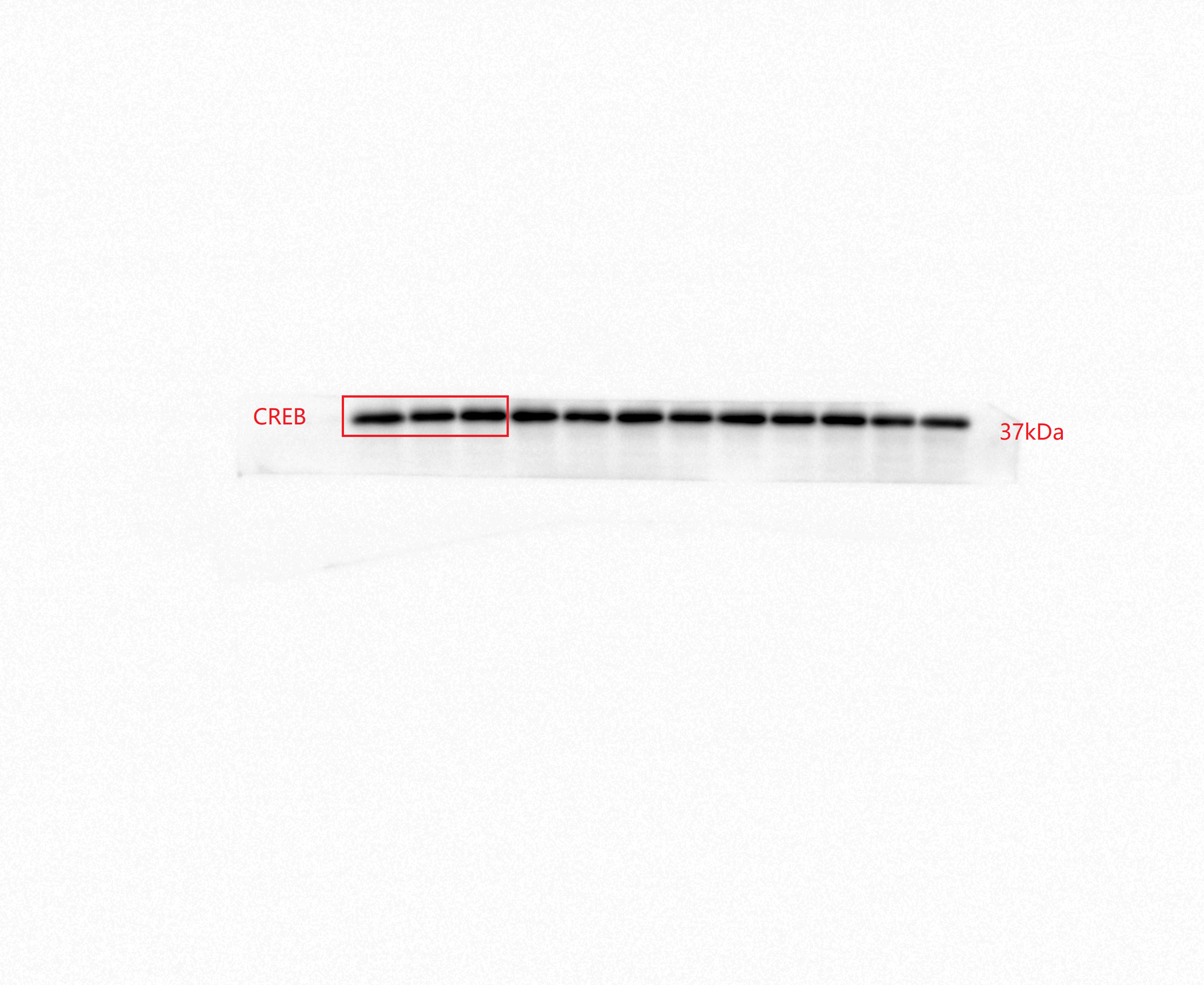

Supplement: Supplementary file 1 — Additional file 1. [file 12902_2022_1086_MOESM1_ESM.zip › CREB.tif]

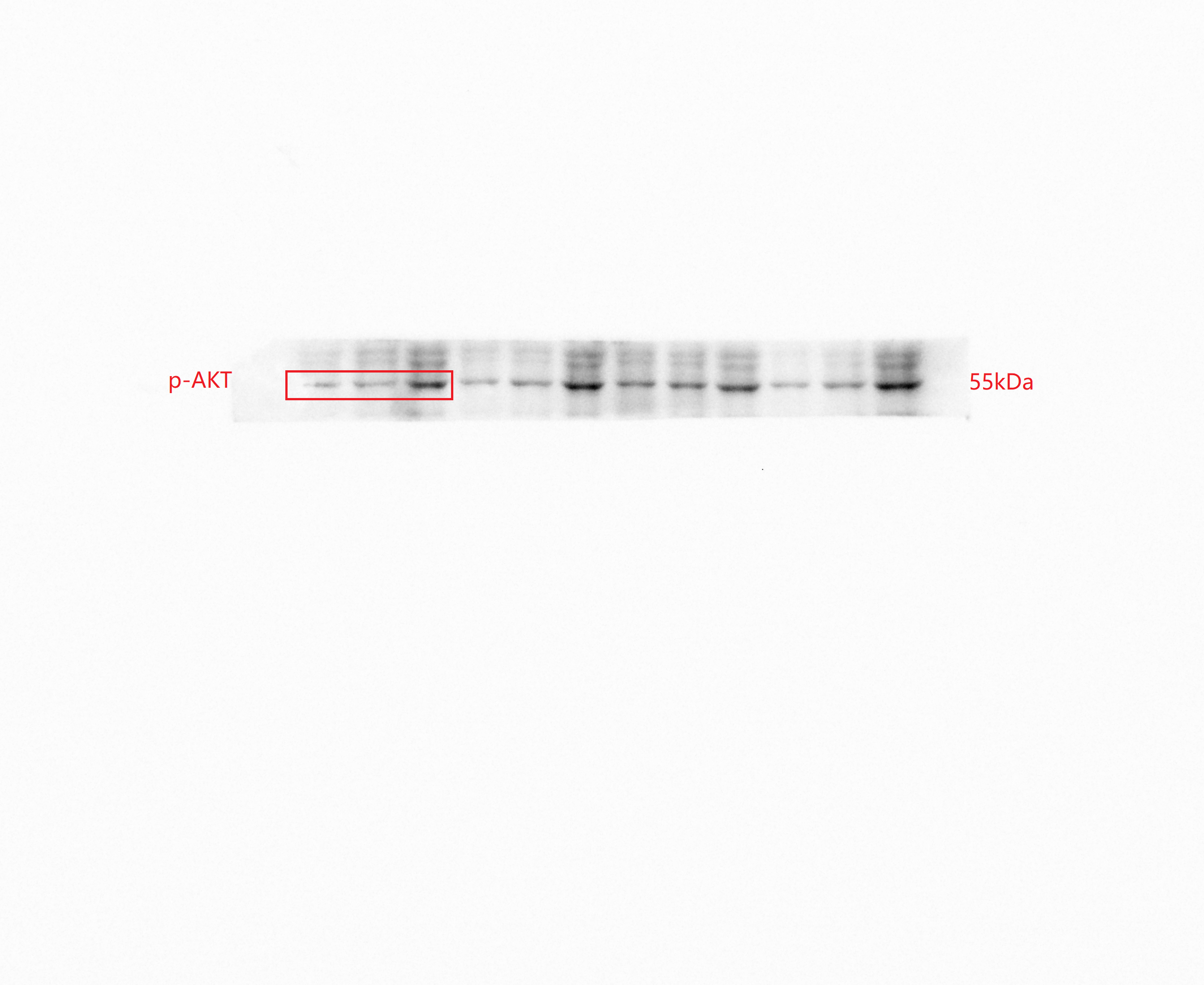

Supplement: Supplementary file 1 — Additional file 1. [file 12902_2022_1086_MOESM1_ESM.zip › p-AKT.tif]

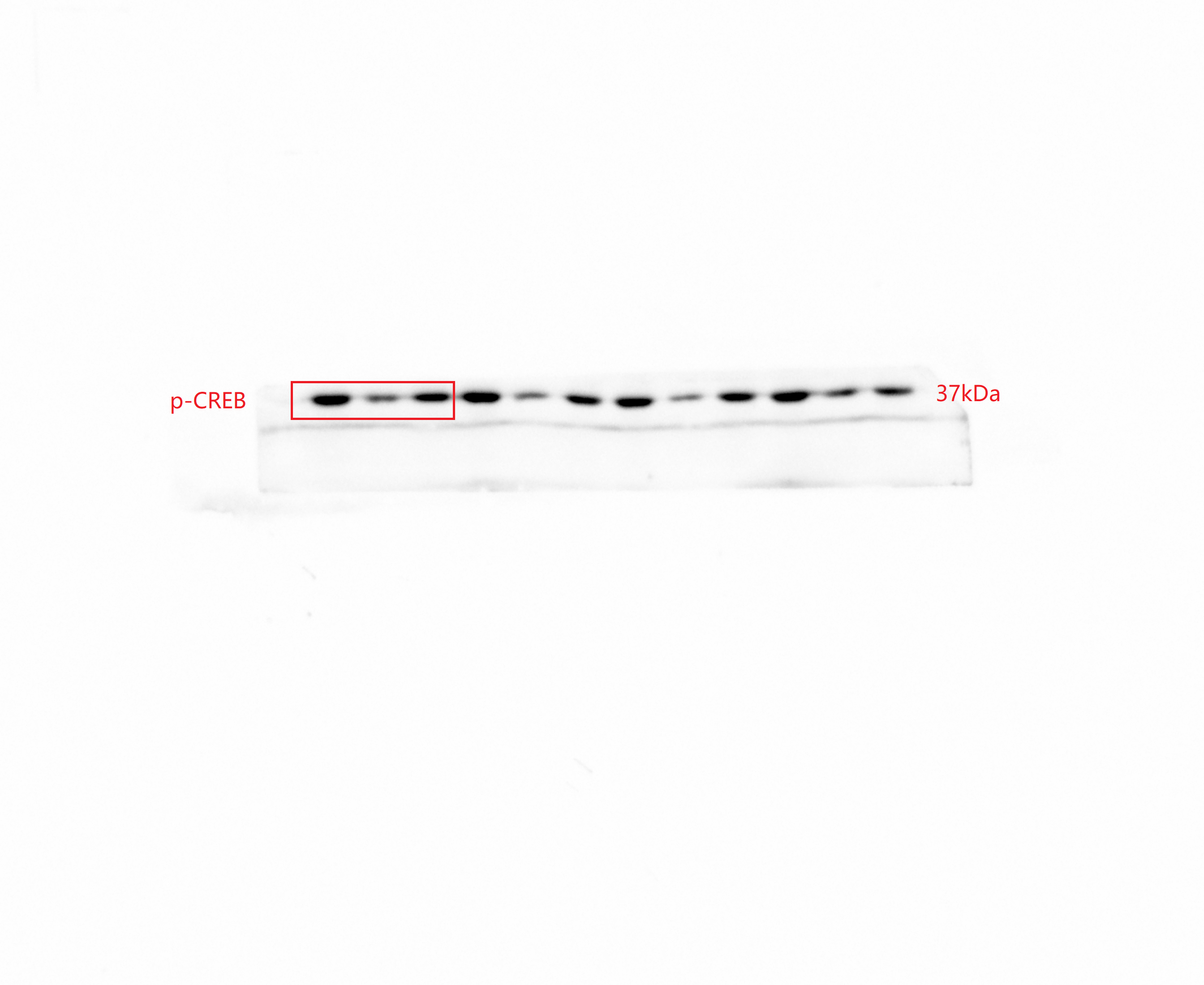

Supplement: Supplementary file 1 — Additional file 1. [file 12902_2022_1086_MOESM1_ESM.zip › p-CREB.tif]

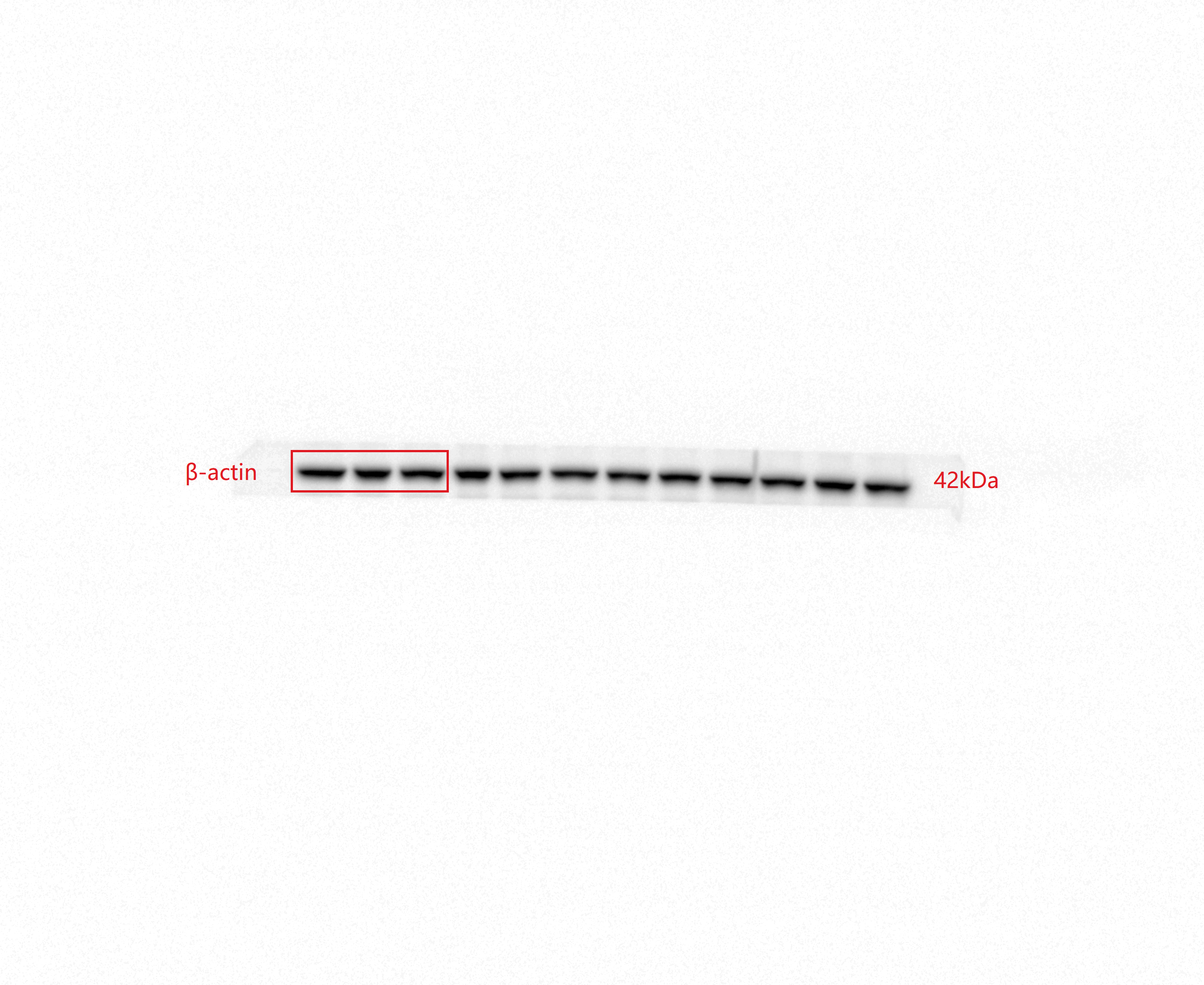

Supplement: Supplementary file 1 — Additional file 1. [file 12902_2022_1086_MOESM1_ESM.zip › Supplementary Information file-original blot of Figure 6C-beta-actin (revise 2).tif]
